# Awareness, Practices, and Demands of Traditional Medicine Providers for Continuous Medical Education in District Hospitals of Vietnam

**DOI:** 10.1155/2020/9852969

**Published:** 2020-06-28

**Authors:** Trung T. Nguyen, Quang N. Nguyen, Dung V. Truong, Tam T. Ngo, Ha N. Vu, Tuan D. Mac

**Affiliations:** ^1^VNU School of Medicine and Pharmacy, Vietnam National University, Hanoi 100000, Vietnam; ^**2**^ Ministry of Health, Hanoi 100000, Vietnam; ^3^Faculty of Health Sciences, Thang Long University, Hanoi 100000, Vietnam

## Abstract

Expanding traditional medicine (TM) coverage in health care is a priority in Vietnam. Continuous medical education (CME) plays an important role in ensuring the quality of TM. However, evidence about TM CME in TM practitioners in Vietnam is insufficient. This paper aimed to evaluate the awareness, practice, and demands on TM CME among TM providers in district hospitals of Vietnam. This cross-sectional descriptive study was performed at the district level at TM hospitals and TM departments of general hospitals in Thanh Hoa Province. Demographic characteristics, awareness, practice, and demand for TM CME were collected via face-to-face interviews. Descriptive statistics and multivariable logistic regression models were applied to examine the factors associated with awareness, practice, and demand for TM CME. The majority of the respondents had ever heard of TM CME (87.5%). Only 60% received TM training in the last five years. Most respondents had a demand for CME (86.8%). The non-Kinh ethnic group (OR = 0.2, 95% CI: 0.1–0.8) and people who had a temporary contract (OR = 0.2, 95% CI: 0.1–0.7) were less likely to be ever heard about TM CME. Higher levels of education (college, OR = 14.1, 95% CI = 1.0–195.9; undergraduate, OR = 9.1, 95% CI = 1.9–44.6) are more likely to be ever heard of TM CME than the vocational training group. Those who regularly update their knowledge are more likely to have heard about TM CME (OR = 7.7, 95% CI = 2.8–21.7) and are more likely to have demands on TM CME (OR = 3.7, 95% CI = 1.2–11.5). Those who had heard about TM CME were more likely to take these courses in the last five years (OR = 6.9, 95% CI = 2.5–18.8). However, this result was the opposite for people with more years of experience (OR = 0.9, 95% CI: 0.8–0.9). There were limited awareness and participation in TM CME but was a high need for CME among TM providers at district hospitals in Vietnam. Promoting lifelong learning and providing promptly supports would be potential to increase the TM CME demands and participation among TM providers.

## 1. Introduction

Traditional medicine (TM) has been encouraged to develop and become increasingly popular worldwide [1–3]. In many countries, such as India, China, and many other parts of Asia, TM can be concerned as folk medicine or alternative medicine [4]. TM practitioners may have undergone formal training or have accumulated only folk experiences or inherit heirloom remedies. Consequently, there is no uniformity in qualifications among TM practitioners. According to the World Health Organization (WHO), a large proportion of TM practitioners have low educational levels [5]. In addition, many TM workers received inadequate training for their practices [6]. Since using natural herbs has many potential risks associated with adverse reactions or drug interactions [7, 8], TM practice cannot solely rely on the traditional experience or beliefs [9]. Therefore, all of TM workers, including those who had undergone formal training, also need to update their knowledge or undergo additional intensive training regularly.

In developing countries such as Vietnam, TM is an essential part of the health system, actively contributing to primary health care, especially among low-income groups. Vietnam has a long history of TM [10], and the Vietnamese government has also set goals and solutions to develop TM widely. According to the plan by 2025, 95% of grassroots health facilities will have TM divisions, and the rate of TM examination and treatment will reach 25% and 30% of total examination and treatment at the district and commune health facilities, respectively [11]. However, statistics from the Vietnam Administration of Traditional Medicine showed that the proportion of TM physicians only accounted for 7.94% in the total number of Vietnamese physicians. The percentage of TM providers with specialized qualifications has not yet reached 6% of the general health workforce [12]. Thus, to achieve the goal of developing the TM system, while ensuring and improving the quality of treatment, continuous medical education (CME) for TM workers is critical besides training new human resources.

CME refers to “a specific form of continuing education, which helps those in the medical field maintain competence and learn about new and developing areas of their field” [13]. In many countries, regular participation in CME is a mandatory requirement for medical practitioners [14–17]. Although CME has been shown to have a positive impact on improving the knowledge, attitudes, skills, behaviors of health workers, and clinical outcomes [14, 18, 19], evidence is needed to design effective TM CME programs. This paper aimed to evaluate the awareness, practice, and demands on TM CME among TM providers in district hospitals of Vietnam. The results will serve to develop interventions or help to inform future research directions into TM CME in Vietnam.

## 2. Materials and Methods

### 2.1. Study Design

This cross-sectional descriptive study was performed at the district level at TM departments of 27 general hospitals and health care facilities in Thanh Hoa Province from January to December in 2017. TM providers were eligible if they (1) worked in the selected hospitals at least six months and (2) agreed to participate in the study. A total of 280TM employees were recruited into the study, including 62TM physicians. People involved in the study had face-to-face interviews and answered a questionnaire designed with four parts: (1) individual characteristics, (2) awareness of TM CME, (3) practices on TM CME, and (4) demand for TM CME. The investigators were medical students of Hanoi Medical University who had been carefully trained in the questionnaire and interview skills. After introducing the research objectives and confirming the providers' participation, the investigators interviewed participants according to the survey in a separate room. Participation in research was entirely voluntary, and the study participants were informed that they could withdraw their participation at any time without influences on their current jobs. The study protocol was approved by the institutional review board of Hanoi Medical University (Code: 45/HĐĐĐĐHYHN).

### 2.2. Variables and Measurement

In this study, we gathered information with a four-part questionnaire including general information and information related to continuing medical education programs; information on short courses includes questions about perceptions, practices, and demands of participants.

1. *Demographic Characteristics.* These included age, gender, ethnicity, educational level, professionals, years of experience, and type of employment contract.
2. *Awareness of TM CME.* We asked participants to report whether they ever heard about TM CME, type of CME they knew, total required CME hours per year according to the Ministry of Health's requirement, whether they updated professional knowledge regularly, and source of knowledge.
3. *Practic*e *on TM CME*. Regarding practice, participants were asked to report whether they had previous CME experience in the last five years (in TM training/general examination and treatment/disease prevention/health programs), total times of previous CME in the last five years, whether they received CME in the central hospital in the last five years, reasons for participating in CME, and barriers of CME participation.
4. *Demand for TM CME*. TM providers were asked to express whether they had demands for TM CME. Moreover, we asked them to report their preference for TM CME, including content, type of training, duration, places of training, and support needed.

### 2.3. Statistical Analysis

Data were imported into the EpiData software with QES., CHEK., and REC. files to limit errors. We used the Stata, version 14.0, software (Stata Corp LP, College Station, Texas, US) to analyze the data. Descriptive statistics and multivariable logistic regression models were applied in this study. The regression models were used to examine the associated factors: (1) heard about TM CME (Yes/No); (2) participate in TM CME in the last five years (Yes/No); and (3) having demands for TM CME (Yes/No). Statistical significance was determined as *p* < 0.05.

## 3. Results

Table 1 shows that our sample had an average age of 34.8 years (SD = 8.6). More than half of them had vocational training education (55.67%). Physician assistants were the most common position (34.3%), followed by TM doctors (22.1%) and nurses. 76.4% of the samples had permanent contracts at the hospital. The mean year of experience was 10.7 (SD = 8.7) years.

Table 2 shows that the majority of the respondents had ever heard about TM CME (87.5%). More than half of TM providers knew the type of CME as short training class (76.8%), and just over one-fifth of people knew the other form of CME, such as seminars, conferences, and workshops (22.9%). About half of the samples thought that a CME must last a minimum of 48 hours every 2 years (55.4%). Most of the respondents regularly updated their knowledge about TM (88.2%), through self-study (72.5%) or attending training courses (56.8%).

According to Table 3, in the last five years, the CME courses that took part in were mainly in health programs (81.4%), general examination and treatment (72.9%), disease prevention (63.9%), or TM training (60.0%). The rate of receiving CME in the central hospital was 47.1%. The hospital's request was one of the main reasons leading to CME participation (51.4%). The respondents said that the barrier of CME participation was the lack of funding (32.1%), lack of time (28.9%), and lack of professional materials (27.5%).

Table 4 shows that most respondents had the demand for CME (86.8%). They wanted to be trained in acupuncture (58.0%), traditional medicine (54.3%), pathology of diseases (48.6%), and nursing, massage, and reflexology skills (42.54%). The most expected form of CME was regular short training classes (78.6%). The majority of the people hoped that the CME courses lasted at least less than one week (63.8%) and held at the hospital where they worked (61.3%). Finance (72.4%), time (50.6%), materials (63.4%), and qualified lecturers (57.2%) are the support most respondents were expected to receive.

In Table 5, we present the relationship between the characteristics of samples with awareness, practice, and demand for TM CME. The non-Kinh ethnic group (OR = 0.2, 95% CI: 0.1–0.8) and people who had a temporary contract (OR = 0.2, 95% CI: 0.1–0.7) were less likely to be ever heard about TM CME. Higher levels of education (college, OR = 14.1, 95% CI = 1.0–195.9; undergraduate, OR = 9.1; 95% CI = 1.9–44.6) are more likely to be ever heard of TM CME than the vocational training group. Those who regularly update their knowledge are more likely to have heard about TM CME (OR = 7.7, 95% CI = 2.8–21.7) and are more likely to have demands on TM CME (OR = 3.7, 95% CI = 1.2–11.5). Those who had heard about TM CME were more likely to take these courses in the last five years (OR = 6.9, 95% CI = 2.5–18.8). However, this result was the opposite for people with more years of experience (OR = 0.9, 95% CI: 0.8–0.9).

## 4. Discussion

This study indicated a moderately low rate of TM providers at district hospitals participated in the TM CME in the last five years, but they had a high demand for training. Findings also show that there were differences in TM CME participation by ethnicity, education, type of contract, and years of experience. Also, disseminating the role of CME and promoting lifelong learning process helped to increase demand for TM CME among health workers. This study suggested the design of future TM CME courses.

In Vietnam, the Law on Examination and Treatment requires a health worker must complete at least 48 hours of CME every two years for maintaining a valid practice license [17]. In this study, only 47.1% of the TM staff receiving CME in the central hospital in the last five years. Although this rate is still higher than a previous report, this result shows that a part of TM workers did not meet the current regulations [17, 20]. Research on the status of human resources and the need for continuous training for TM providers in the Thai Binh province, Vietnam, showed that only 36% of them had participated in CME [20]. The absence of a mechanism to review and assess compliance, along with the fact that the CME is mostly implemented at the central and provincial levels, is the main reason for the lack of opportunities for district and commune health workers to participate in the CME [21]. The results also show that participation is closely related to having heard about TM CME. However, the regression results indicate the discrepancies in the awareness of TM CME, since ethnic minorities, who have intermediate education, term contracts, were less aware of TM CME than others. This result suggests that interventions are needed to increase the awareness of TM providers about CME, especially in these groups.

The demand for joining CME of TM officers was 86.8%, which was higher than a previous study (68–80%) [22]. The demand on continuous training was also quite high among other health professionals in Vietnam. For instance, 97.2% of medical staff in private maxillofacial clinics in Thai Binh expect to receive continuous training [23]. Another study suggested that all village health workers in the Nam Dinh province showed their demands on CME participation [24]. A similar result was also found in a North Carolina study, which indicated that there was a low access but high demand on cancer-related CME topics among primary care physicians [25]. In fact, effective CME programs require appropriate formulation and actual needs [26]. This study reported a high percentage of TM employees having demands on TM CME, indicating the feasibility to organize TM CME programs in district levels of the Vietnam health system. However, findings also show that after adjusting for potential confounders, TM providers who regularly updated their knowledge were more likely to have a higher demand for CME participation. Knowledge updating, skills training, and capacity building are essential to meet the health care needs of patients. Therefore, the policy makers should perform more educational campaigns, in addition to tightening the implementation of the regulations on the time to join the CME, to promote lifelong learning of health workers.

The study also suggests issues related to the organization of CME. The TM CME programs created must ensure quality and meet the academic needs of the TM staff. CME with the content of acupuncture, traditional medicine, and pathology of diseases should be encouraged at district hospitals in Thai Binh. TM providers also preferred short courses that lasted less than one week and were held at the places where they worked. In addition, course organizers might strive to provide support to remove barriers when TM providers were involved in CME. In this study, difficulties were reported, including lack of time, money, and materials. Besides, the lack of appropriate CME programs, workload issues, long distance, and the permission of the leaders were the reasons why medical staff had difficulty in registering CME courses [27–29]. However, it is difficult to solve these issues completely without the participation of national regulations and policies.

To date, this is one of the few studies evaluating the current situation and the continuing education needs of TM providers in Vietnam. This survey shows the need for ongoing training and suggestions for developing further interventions. Nonetheless, some limitations need to be noted. Our sample only included TM providers at the district hospitals in one province, which might not reflect the CME situations of TM providers in other levels of health system or in other provinces. Besides, the local study site in one province was not representative of TM providers in entire Vietnam.

## 5. Conclusions

There were limited awareness and participation in TM CME but a high need for CME among TM providers at district hospitals in Vietnam. Promoting lifelong learning and providing promptly supports would be potential to increase the TM CME demands and participation among TM providers.

## Figures and Tables

**Table 1:** Demographic characteristics of respondents.

Characteristics	*n*	%
Gender, Male	98	35.0
Ethnic, Kinh	259	92.5
Education		
Vocational training	156	55.7
College	33	11.8
Undergraduate	66	23.6
Postgraduate	25	8.9
Professionals		
Traditional medicine physicians	62	22.1
Bachelor of nurse	30	10.7
Practical nurse	35	12.5
Assistant physicians	96	34.3
Others	57	19.4
Years of experience		
1–5 years	100	35.7
6–10 years	63	22.5
11–15 years	60	21.4
>15 years	57	20.4
Type of contract		
Permanent	214	76.4
Temporary	61	21.8
Others	5	1.8
	Mean	SD
Age (years)	34.8	8.6
Years of experience (years)	10.7	8.7

**Table 2:** Awareness about CME on TM among participants.

Characteristics	*n*	%
Ever heard about TM CME	245	87.5
Type of CME		
Short training class	215	76.8
Seminar, conference, workshop	64	22.9
Do not know	45	16.1
Total required CME hours		
48 hours every 2 years	155	55.4
120 hours every 5 years	48	17.1
>0 hours	35	12.5
Do not know	42	15.0
Update professional knowledge frequently	247	88.2
Source of knowledge		
Self-learning	203	72.5
Training	159	56.8
Others	11	3.8
Do not know	29	10.4

**Table 3:** Practices in CME on TM among participants.

Characteristics	*n*	%
Previous CME experience in the last five years		
TM training	168	60.0
General examination and treatment	204	72.9
Disease prevention	179	63.9
Health programs	228	81.4
Total time of previous CME in the last five years		
<3 days	48	17.2
3–6 days	33	11.8
7–12 days	76	27.1
>12 days	79	28.2
Do not know	44	15.7
Receive CME in the central hospital in the last five years	132	47.1
Reasons for participating in CME		
Assignment from hospital	144	51.4
Self-training	120	42.9
Barriers of CME participation		
Lack of time	81	28.9
Lack of funding source	90	32.1
Lack of materials	77	27.5
Hospital did not allow to participate	1	0.4
Other	14	5.0
None	112	40.0

**Table 4:** Demands on TM CME.

Characteristics		*n*	%
Have demand for TM CME (*n* = 280)		243	86.8
Content of TM CME (*n* = 243)			
Fundamental knowledge		79	32.5
Acupuncture		141	58.0
Pathology of diseases		118	48.6
Traditional medication use		132	54.3
Nursing, massage, and reflexology skills		103	42.4
Others		10	4.1
Training type (*n* = 243)			
Regular short training classes		191	78.6
Long-term training classes		50	20.6
Retraining to change professionals		9	3.7
Others		6	2.5
Duration of each training class (*n* = 243)			
<3 days		78	32.1
3–6 days		77	31.7
>6 days		88	36.2
Places of training class (*n* = 243)			
Hospitals where participants worked		149	61.3
Hospitals in higher levels of system		50	20.6
Training organizations		48	19.8
Support needed			
Financial		176	72.4
Time	123	50.6
Materials	154	63.4
Qualified lecturers	139	57.2
TM knowledge	97	39.9
TM practice	85	35.0

**Table 5:** Factors associated with awareness, practice, and demand for TM CME.

Characteristics	Heard about TM CME	Participated in TM CME in the last five years	Had demands on TM CME
	OR (95% CI)	OR (95% CI)	OR (95% CI)
Age	0.9 (0.9–1.1)	1.1 (1.0–1.2)^*∗*^	0.9 (0.8–1.0)
Gender			
Female	1	1	1
Male	0.9 (0.3–2.2)	1.1 (0.6–1.9)	0.6 (0.3–1.5)
Ethnic			
Kinh	1	1	1
Others	0.2 (0.1–0.8)^*∗*^	0.5 (0.2–1.6)	1.4 (0.3–6.4)
Education			
Vocational training	1	1	1
College	14.1 (1.0–195.9)^*∗*^	0.5 (0.1–2.3)	0.4 (0.1–3.5)
Undergraduate	9.1 (1.9–44.6)^*∗*^	0.6 (0.2–1.6)	2.2 (0.6–8.0)
Postgraduate	-	0.9 (0.2–3.6)	3.8 (0.7–21.4)
Professionals			
Traditional medicine physicians	1	1	1
Bachelor of nurse	0.6 (0.0–7.4)	1.3 (0.2–8.1)	7.9 (0.6–3.5)
Practical nurse	3.7 (0.7–19.8)	1.8 (0.5–6.7)	4.6 (0.7–8.0)
Assistant physicians	5.7 (1.2–26.7)^*∗*^	0.6 (0.2–2.0)	4.1 (0.9–18.6)
Others	3.9 (0.7–21.9)	1.1 (0.4–3.0)	0.8 (0.2–2.9)
Years of experience (years)	0.9 (0.8–1.1)	0.9 (0.8–0.9)^*∗*^	1.0 (0.9–1.1)
Type of contract			
Permanent	1	1	1
Temporary	0.2 (0.1–0.7)^*∗*^	0.7 (0.4–1.4)	0.4 (0.1–1.1)
Others	0.1 (0.0–2.2)	0.9 (0.0–4.4)	-
Update professional knowledge frequently			
No	1	1	1
Yes	7.7 (2.8–21.7)^*∗*^	2.4 (0.9–6.1)	3.7 (1.2–11.5)^*∗*^
Ever heard about TM CME			
No		1	1
Yes		6.9 (2.5–18.8)^*∗*^	1.9 (0.6–6.8)
Participate in TM CME in the last five years			
No			1
Yes			0.9 (0.3–2.2)

^*∗*^ *p* < 0.05.

## Data Availability

The data used to support the findings of this study are available from the corresponding author. Requests for access to individual subject data may be made to Trung T. Nguyen (thanhtrungnguyen.smp@gmail.com).

## Supplementary Materials

Supplementary Materials Questionnaire used in this study.
