# Supplementary material for: Awareness, Practices, and Demands of Traditional Medicine Providers for Continuous Medical Education in District Hospitals of Vietnam
Source: Evid Based Complement Alternat Med. 2020 Jun 28;2020:9852969. doi: 10.1155/2020/9852969 (PMC7341395; doi:10.1155/2020/9852969)
Supplement: Supplementary Materials — Questionnaire used in this study. [file 9852969.f1.pdf]

APPENDIX
THE QUESTIONNAIRE FOR TRADITIONAL MEDICINE STAFF

Code: ……………………………………………………

Date of investigation:………………………………

Supervisor:…………………………………………..

Dear..............................,

We invite you to take part in the CME status survey for TM medical staff. The results of the survey will provide useful information for health managers in organizing CME programs or creating employee support packages. Your personal information will be kept entirely confidential.

Are you willing to participate in this survey?

1. *No → End*
2. *Yes→ Please complete the following information.*

A. GENERAL INFORMATION

A1. Name:……………………………………………….

A2. Age: …………………………………………

A3. Gender: □ *Male* □ *Female*

A4. Ethnic: ………………………………………

A5. Education: ………………………………………

A6. Qualification:

□ *General Practitioner* □ *Medico*

□ *Traditional medicine physicians* □ *Specialist level I*

□ *Bachelor of Pharmacy* □ *Specialist level II*

□ *College pharmacist* □ *Master*

□ *Range pharmacist* □ *Doctor*

□ *Bachelor of nursing* □ *Khác*

□ *College nursing*

□ *Vocational Nursing*

A7. Training places:……………………………………………………………..

A8. Workplace after graduation*:*…………………………………………......

A9. Current workplace:……………………………………………………….

A10. Years of experience ……………………………………….*years*

A11. Type of contract: □ *Permanent* □ *Temporary* □ *Other*

B. THE PROCESS OF CONTINUOUS TRAINING AND SHORT-TERM TRAINING ON TRADITIONAL MEDICINE

B1. Have you ever heard of Traditional Medicine (TM) Continuous Medical Education (CME)?

□ *Yes* □ *No*

B2. In your opinion, what types of CME are there?

□ *Short training class*

□ *Seminar, conference, workshop*

□ *Others,…………………………………………………………………..*

B3. According to you, who is responsible for participating in CME?

□ *Doctor, university pharmacist or above*

□ *The only medical staff of secondary, intermediate and college levels*

□ *Health workers working in public units*

□ *Health workers working in private units*

□ *All health workers working in medical units*

B4. In your opinion, how long is the required time to attend the CME?

□ *48 hour every 2 years*

□ *120 hours every 5 years*

□ *Just join in, as long as you like*

□ *Don't know*

B5. Have you updated your knowledge about TM during your working period?

□ *Yes → B5x1* □ *No → B7*

B5x1. If yes, from what source do you update your knowledge?

□ *Self – learning*

□ *Training*

□ *Others: ....*

B5x2. Which courses did you take?

□ *Participate in short-term courses with certificates related to TM*

□ *Attend training courses related to medical examination and treatment*

□ *Attend short-term training courses related to prevention*

□ *Attend short-term training courses related to health programs and projects*

B5x2a. How many training days have you taken during your working period?..........................*days*

B5x2b. Were you guided by a doctor from a higher level hospital during your working period in the hospital?

□ *Yes* □ *No*

B5x2c. Dìd your hospital has a doctor from the higher-level hospital who has strengthened to do the medical examination?

□ *Yes* □ *No*

B5x2d. Can you go to the central hospital to learn more?

□ *Yes* □ *No*

B5x2e. What is the form of study (if any)?..

B5x3. During 2013 - 2018, how many times did your hospital organize staff training?.........*lần*

B5x4. During 2013-2018, how many short-term courses or training did you receive?....*courses*

**No.**	**Course name**<br><br>*(Please specify)*	**Year**<br><br>*(Please specify)*	**Place**<br><br>*1. Hospital where you work*<br>*2. Hospital of upper level*<br>*3. Educational institution*	***During (days)***<br><br>*(Please specify*
1	....	....	....	....
2	....	....	....	....
3	....	....	....	....
4	....	....	....	....
5	....	....	....	....
6	....	....	....	....
7	....	....	....	....
8	....	....	....	....
9	....	....	....	....

B5x5. Why do you train, update knowledge?

☐ *State requirements, place of work*

☐ *Personal wishes for updates to improve proficiency*

☐ *Others (Please specify):* ....

B6. Do you have any difficulty in updating knowledge about TM?

□ *Cannot arrange a time*

□ *Lack of funding*

□ *Lack of funding*

□ *Others (Please specify):* ....

B7. In the future, do you need to update your knowledge about TM?

□ *Yes → B7x1* □ *No → END*

B7x1. What do you aspire to learn about?

□ *Fundamental knowledge*

□ *Acupuncture*

□ *Pathology of diseases*

□ *Traditional medication use*

□ *Nursing, massage, reflexology skills*

□ *Others,..*

B7x2. How many courses do you want in a year?*..................courses.*

B7x3. What kind of course do you want to take?

□ *Regular training by year*

□ *Learn to concentrate*

□ *Retrain*

B7x4. How many days do you want to attend the course?*..........................days*

B7x5. Where would you like the course to be held?

□ *Hospital where you work*

□ *Hospital of upper level*

□ *Educational institution*

B7x6. You want to attend the course organized by any unit?

□ *Hospital where you work*

□ *Hospital of upper level*

□ *Ministry of Health*

□ *Educational institution*

□ *Projects*

B8. Do you have any suggestions when attending short-term training in TM?

□ *Support for training expenses*

□ *Support for time to study*

□ *Support for professional documentation*

□ *Lecturers from higher schools or hospitals*

□ *Support on TM knowledge*

□ *Support on TM practice*

□ *Other assistance (please specify):...*

Thank you!
